# Bacteriological Profile of Diabetic Foot Ulcers and Detection of Methicillin-Resistant Staphylococcus aureus and Extended-Spectrum β-Lactamase Producers in a Tertiary Care Hospital

**DOI:** 10.7759/cureus.20596

**Published:** 2021-12-22

**Authors:** Srinath Selvarajan, Sarumathi Dhandapani, Anuradha R., Lavanya T., Anandhi Lakshmanan

**Affiliations:** 1 General Practice, Government Kiplauk Medical College, Chennai, IND; 2 Microbiology, ESIC Medical College and Post Graduate Institute of Medical Sciences and Research, Chennai, IND; 3 Community Medicine, ESIC Medical College and Post Graduate Institute of Medical Sciences and Research, Chennai, IND; 4 Microbiology, Government Kiplauk Medical College, Chennai, IND

**Keywords:** phenotypic test, antimicrobial therapy, esbl, mrsa, diabetic foot infection

## Abstract

Introduction

Diabetic foot infection is the most dreaded complication of diabetes mellitus and the commonest cause of hospitalization and limb amputation. Identification of the causative agent responsible for diabetic foot infection and the earliest initiation of appropriate antimicrobial therapy are vital for the control and prevention of the complication of diabetic foot ulcers. Therefore, we conducted this study to determine the bacteriological profile of diabetic foot ulcers and to detect methicillin-resistant *Staphylococcus aureus* (MRSA) and extended-spectrum β-lactamase (ESBL) producers in our institute.

Methodology

During the study period, samples were collected from the foot ulcers of 100 patients at the Diabetic Outpatient Department. The samples were processed according to the standard laboratory protocol, and bacterial isolates were identified. Antibiotic susceptibility testing was performed using the modified Kirby-Bauer disk diffusion technique, and results were interpreted according to the Clinical and Laboratory Standards Institute guidelines (CLSI 2016). A phenotypic test for MRSA detection was performed using cefoxitin (30 μg) disk.

Results

The highest incidence of diabetic foot ulcers was observed in patients aged 41-50 years. There were 83 men and 17 women, with a male to female ratio of 4.882. Of the 100 collected samples, 73 were positive for microbial growth, and 27 samples showed no growth. Of the 73 positive cultures, monomicrobial infection was found in 48 patients, and polymicrobial infection was found in 25 patients. Gram-positive pathogens were isolated from 34 patients, and gram-negative microbes were isolated from 64 patients. Among all collected isolates (n=100), *Staphylococcus aureus* was the most predominant organism and *Acinetobacter* species was the least common (only two isolates). Among the gram-negative bacteria, *Pseudomonas aeruginosa* was predominant. All the isolated gram-positive bacteria were susceptible to vancomycin. Gram-negative bacteria were highly susceptible to colistin with the exception of *Proteus* species which is intrinsically resistant to colistin and it is not reported for *Proteus* species. ESBL producers were primarily found among* Klebsiella *species isolates (22.22%). Among 29 *S. aureus *isolates, 8 (27.5%) were found to be MRSA producers.

Conclusion

Based on the bacteriological profile of diabetic foot ulcers, *S. aureus *among the gram-positive isolates and *P. aeruginosa *among the gram-negative isolates were the predominant pathogens. Infections caused by multidrug-resistant bacteria such as MRSA and ESBL producers have been reported with increasing frequency. According to the antibiotic susceptibility pattern, treatment can be initiated, continued, or altered, thereby reducing morbidity in patients with diabetic foot ulcers.

## Introduction

Diabetes is recognized as one of the four priority noncommunicable diseases targeted for action by the United Nations as a result of the growing disease burden [1,2]. The total number of people with diabetes has been estimated to increase from 171 million in 2000 to 366 million in 2030 [3]. This may be contributed by population growth, aging, urbanization, and growing obesity issues [3,4]. Type 2 diabetes accounts for the majority (>85%) of the total diabetes prevalence. Diabetic foot infection is the most dreaded complication of diabetes mellitus and one of the commonest causes of hospitalization among patients with diabetes [5,6]. Patients with diabetes suffer from loss of sensation due to neuropathy, which leads to sensory loss commonly in the lower extremities, and hence they sustain injuries. These patients are susceptible to foot ulcers due to three primary conditions, viz., neuropathy, peripheral arterial disease, and pressure overload. This situation leads to the loss of protective sensation, foot deformities, gait disorders, anterior displacement of weight-bearing during walking [7], and reduced mobility. These problems are generally accompanied by arterial insufficiency. The most serious consequence of diabetic foot ulcers is limb amputation, which occurs 10-30 times more frequently in patients with diabetes than in the general population [8]. Diabetic foot infections commonly occur due to various bacterial infections. Therefore, identification of the causative organism and selection of appropriate antibiotics are vital for the management of diabetic foot ulcers. Multidrug-resistant bacteria, methicillin-resistant *Staphylococcus aureus* (MRSA), and extended-spectrum β-lactamase (ESBL)-producing gram-negative bacteria and their associated complications have resulted in a huge health concern among medical and clinical practitioners. An antibiotic susceptibility test is required to select the correct therapeutic agent for the management of these bacteria. Accurate identification of the causative agent and the earliest initiation of appropriate antimicrobial therapy comprise an essential component of the control and prevention of the complication of diabetic foot ulcers. Therefore, we conducted this study to determine the bacteriological profile of diabetic foot ulcers and to detect MRSA and ESBL producers in our institute.

## Materials and methods

Study design

This research was a descriptive study conducted during a period of three months at the Department of Microbiology, Government Kilpauk Medical College and Hospital located in South India. The study was approved by the Indian Council of Medical Research (ICMR), New Delhi and the approval reference ID is 2017-07099.

Inclusion criteria

Patients with type 1 and type 2 diabetes of all age groups and both sexes visiting the Diabetes Outpatient Department with foot ulcers of various grades were included in this study.

Exclusion criteria

Patients with foot ulcers due to causes other than diabetes such as trauma and those visiting other outpatient departments were excluded. Patients with diabetic foot ulcers who were severely ill and unable to provide informed consent were excluded.

Data collection

Samples were collected from 100 patients visiting the Outpatient Department with diabetic foot ulcers. The data collection was performed using a standard proforma. Informed consent was obtained from all patients, and their confidentiality and safety were maintained.

Sample collection and processing

Samples were collected from the deeper portion or base (deep swab technique) of the diabetic foot ulcer from the 100 patients using two sterile swabs, which were dipped in a sterile broth. The samples were collected by making a firm, rotatory movement with the swabs. The ulcer was debrided before sampling with a sterile scalpel and rinsed with sterile normal saline, and then the samples were collected to prevent contamination with colonizing bacteria. One swab was used for gram staining, and the other was used for culture. A direct gram-stained smear of the specimen was examined. The specimens were inoculated onto blood agar, MacConkey agar, and nutrient agar and incubated aerobically at 37°C overnight and then the plates were examined for growth. If no microbial growth was found at the end of 48 hours, the culture report was given as 'no growth '. Further processing was done according to the nature of the isolate, which was identified based on gram staining, colony morphology, and biochemical characteristics. The bacterial isolates were lawn cultured on Mueller-Hinton agar for susceptibility testing according to the modified Kirby-Bauer disk diffusion technique. The interpretation was done by measuring the sizes of the zone of inhibition according to the Clinical and Laboratory Standards Institute guidelines (CLSI M100 26th edition). The antimicrobial disks used for gram-negative bacilli were ampicillin (20µg), gentamicin (10µg), amikacin (30µg), cefuroxime (30µg), levofloxacin (5µg), ceftazidime (30µg), cefotaxime (30µg), ceftriaxone (30µg), cefepime (30µg), cefoperazone/sulbactam (75/10µg), piperacillin/tazobactam (100/10µg), meropenem (10µg), and colistin (1µg). Ampicillin (20µg), cefoxitin (30µg), erythromycin (15µg), amikacin (30µg), cotrimoxazole, ciprofloxacin (5µg), levofloxacin (5µg), doxycycline (30µg), vancomycin (30µg), linezolid (30µg), and high-level gentamicin (80µg) were used to examine the susceptibility pattern of gram-positive bacteria. *Escherichia coli* ATCC 25922, *Pseudomonas aeruginosa* ATCC 27853, *S. aureus* ATCC 29213, and *Enterococcus faecium *ATCC 29212 were used as quality control strains. The phenotypic test for the detection of MRSA was performed using a cefoxitin (30 μg) disk. The isolates that produced a zone of inhibition of ≤21 mm were considered as MRSA. ESBL production was confirmed using disks of ceftazidime (30 μg) and ceftazidime/clavulanate (30/10 μg) and also cefotaxime (30 μg) and cefotaxime/clavulanate (30/10 μg). An increase in zone diameter to ≥5 mm for either antimicrobial agent tested in combination with clavulanate vs the zone diameter of the antimicrobial agent when tested alone indicated that the strain was an ESBL producer.

## Results

As shown in Table 1, no isolates were collected from patients aged <30 years. Only one patient >80 years old presented with foot ulcers. Patients in the age group 41-70 visited the diabetic outpatient department with foot ulcers more commonly. The highest prevalence of diabetic foot ulcers, i.e., 34%, was observed in patients in the age group of 41-50 years. This was followed by patients in the age group 51-60 years.

**Table 1:** Age distribution of patients presented with diabetic foot ulcers

Patient age group	Percentage (%)
31 - 40	04
41- 50	34
51- 60	33
61 -70	23
71- 80	05
>80	01

**Figure 1:**
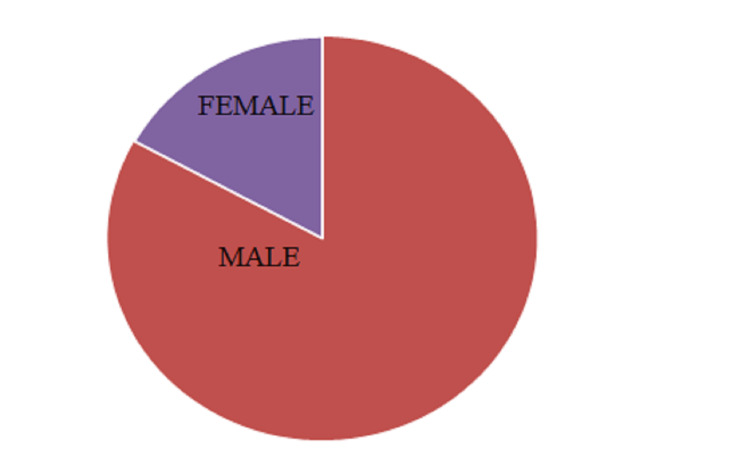
Sex-wise distribution (n=100)

As shown in Figure 1, more male patients visited the diabetic outpatient department with foot ulcers. The total number of males and females was 83 and 17, respectively. This revealed that diabetic foot ulcers were prevalent in the male population in our study.

Of the 100 samples collected, 73 samples showed positive cultures, and 27 samples showed no microbial growth. Of the 73 positive cultures, monomicrobial infection was found in 48 patients, and polymicrobial infection was found in 25 patients [Table 2]. 

**Table 2:** Characteristics of aerobic bacterial culture

S. No.	Characteristics	Number of Specimen/isolates
1	Total number of samples collected	100
2	Culture positive samples	73
3	Culture negative samples	27
4	Gram positive bacteria isolates	34
5	Gram negative bacteria isolates	64
6	Mono microbial infection	48
7	Mono microbial infection with gram positive bacteria	17
8	Mono microbial infection with gram negative bacteria	31
9	Poly microbial infection	25

As tabulated in Table 3, the most predominant isolate was *Staphylococcus aureus *(29%). *Pseudomonas aeruginosa* was the second most prominent among all the collected isolates. *Acinetobacter* species was the least predominant (2%).

**Table 3:** Number of identified gram positive and gram negative organisms

Gram staining property	Organism	Number of isolates
Gram positive	*Staphylococcus aureus*	29
Gram negative	*Pseudomonas aeruginosa*	28
Gram negative	*Klebsiella* species	22
Gram negative	*Proteus* species	07
Gram positive	*Enterococcus* species	05
Gram negative	*Escherichia coli*	05
Gram negative	*Acinetobacter* species	02

As shown in Table 4, all five isolates of *Enterococcus *species showed resistance to doxycycline. *Staphylococcus aureus* showed maximum resistance to cotrimoxazole (13 isolates). 

**Table 4:** Drug resistance of gram positive organisms isolated Numbers within the table denote the number of isolates showing resistance to that particular antibiotic; cefoxitin is used as surrogate marker for detection of methicillin-resistant *Staphylococcus aureus *(MRSA) IR - intrinsic resistance (not tested/not reported); n - total number of isolates

Organism (n)	Amikacin	Gentamicin	Cotrimoxazole	Doxycycline	Erythromycin	Linezolid	Ciprofloxacin	Levofloxacin	Cefoxitin	Vancomycin	High level gentamicin
*Enterococcus *species(5)	0	0	IR	5	4	0	0	0	IR	0	0
*Staphylococcus aureus*(29)	0	9	13	0	9	0	5	9	8	0	Not tested

As shown in Table 5, among two *Acinetobacter* species isolated, one isolate was resistant to gentamicin and sensitive to other antibiotics. Among *Pseudomonas aeruginosa, *which was predominant among gram negative organism, no isolates showed resistance to amoxyclav, piperacillin-tazobactam, ceftazidime, cefotaxime, cefoperazone sulbactam meropenem, levofloxacin, cefepime and colistin. No organism showed resistance to colistin.

**Table 5:** Drug resistance of gram negative organisms Numbers within the table denote the number of isolates showing resistance to that particular antibiotic IR - intrinsic resistance (not tested/not reported); n - number of isolates

Organism	Amikacin	Gentamicin		Ampicillin	Amoxyclav	Piperacillin-tazobactam	Ceftazidime	Cefotaxime	Cefoperazone Sulbactam	Ceftriaxone	Meropenem	Levofloxacin	Cefepime	Cefuroxime	Colistin
*Acinetobacter* species(n=2)	0	1	IR		IR	0	0	0	0	0	0	0	0	IR	0
*Escherichia coli*(n=5)	0	0	1		1	2	1	1	2	1	0	0	0	0	0
*Klebsiella* species(n=22)	4	5	IR		4	6	5	5	0	1	2	2	1	0	0
*Proteus* species(n=7)	0	0	IR		0	0	0	0	2	0	0	0	0	IR	IR
*Pseudomonas aeruginosa *(n=28)	2	5	IR		IR	0	0	0	0	IR	0	0	0	IR	0

As in Table 6, ESBL producers were more prevalent in *Klebsiella* species compared to *Escherichia coli. *ESBL testing was not performed in other organisms.

**Table 6:** Percentage of ESBL producers and MRSA ESBL - extended spectrum beta lactamases; MRSA - methicillin-resistant *Staphylococcus aureus*

Organism	Total isolates	ESBL producers/MRSA	Percentage (%)
*Klebsiella* species	22	5 (ESBL)	22.7
Escherichia coli	5	1 (ESBL)	20
Staphylococcus aureus	29	8 (MRSA)	27.5

## Discussion

Diabetic foot infections rarely present as cellulitis or postsurgical infections, but they are most commonly a consequence of ulcerations secondary to progressive peripheral polyneuropathy [9]. Systemic antibiotics must be initiated as early as possible in patients with clinically infected diabetic foot ulcers, whereas topical antibiotics and antiseptics are not recommended as the only treatment [10,11,12]. Targeted therapy based on antibiotic susceptibility results is essential. Most diabetic foot infections are known to be polymicrobial [13,14]. However, the present study showed more number of monomicrobial infections.

Of the 100 samples collected from patients with diabetic foot ulcers, the maximum number of infections was found in patients aged 41-50 years. In the literature, the maximum number of infections was reported in patients aged 51-60 years by Ibrahim et al. [15] and in patients aged 60-65 years by Shanmugam et al [16]. This may be attributed to the high prevalence of comorbid conditions in this age group. According to our results, diabetic foot infection was more prevalent among men than among women, which is consistent with a study conducted by Anandi et al [17]. The male to female ratio is 4.882:1 in our study. This shows the more prevalence of diabetic foot ulcers in males.

*S. aureus* was found to be the most predominant of all isolates, which is consistent with the findings reported by Vidhani et al. and Zubair et al [18,19]. However, Konar and Das reported the predominance of *P. aeruginosa*, followed by *E. coli* [20]. Gram-positive bacteria were almost susceptible to vancomycin. Similarly, Al Benwan et al. reported that vancomycin was the most effective treatment for gram-positive bacteria, and imipenem, piperacillin/tazobactam, and amikacin were effective against gram-negative infections [21]. We did not test with imipenem. In this study, we have avoided testing and reporting results of certain antibiotics against some bacteria which was known to be intrinsically resistant. This prevented unnecessary usage of antibiotic disks and also this resulted in ending the treatment with such antibiotics for the patients if empirically initiated.

In the present study, ESBL producers were primarily found among *Klebsiella* species (22.22%), a finding that is in contrast to the study of Chavan et al, who reported that *E.coli *(68.3%) showed the highest ESBL activity [22]. Similar to our results, ESBL activity was detected in 30.18% of *K. pneumoniae *species in a study conducted by Shukla et al [23]. Priyadharshini et al reported that 37.5% were ESBL producers [16]. Among the 29 *S. aureus* isolates, eight (27.5%) were found to be MRSA. Similarly, Konar and Das [20] reported 36.8% MRSA. In another study conducted by Vidhani et al, *S. aureus* was isolated from 188 patients (41.8%), of which MRSA constituted 51.6% [18]. MRSA and ESBL detection were helpful in the initiation of targeted therapy. Vancomycin was initiated for patients with MRSA growth and the beta-lactam group of drugs was avoided for patients with ESBL growth. Further, contact precautions can be adapted in such patients to prevent the spread of antimicrobial resistance (AMR).

Limitation of the study

Factors such as occupation, behavioral habits, physical activity, and body mass index, and other comorbidities were not evaluated in our study. Molecular studies were not performed for the confirmation of ESBL producers and MRSA.

## Conclusions

This study on the bacteriological profile of diabetic foot ulcers revealed that *S. aureus* among the gram-positive pathogens and *P. aeruginosa* among the gram-negative pathogens were the predominant microbes. Infections caused by multidrug-resistant bacteria such as MRSA and ESBL producers have been reported with an increasing frequency among diabetic foot infections. If appropriate targeted therapy is not initiated, this may lead to serious consequences such as limb amputation. Understanding the antibiotic sensitivity and resistance pattern would be helpful in determining drugs for treatment, thereby reducing morbidity and mortality in patients with diabetic foot ulcers.
